# All-cause and cause-specific mortality in social anxiety disorder: a matched cohort and sibling cohort study

**DOI:** 10.1017/S2045796026100535

**Published:** 2026-03-27

**Authors:** Lorena Fernández de la Cruz, Kayoko Isomura, Ralf Kuja-Halkola, Josep Pol-Fuster, Zheng Chang, Brian M. D'Onofrio, Henrik Larsson, Paul Lichtenstein, Anna Sidorchuk, David Mataix-Cols

**Affiliations:** 1 Centre for Psychiatry Research, Department of Clinical Neuroscience, Karolinska Institutet & Stockholm Health Care Services, Region Stockholm, Stockholm, Sweden; 2 Department of Medical Epidemiology and Biostatistics, Karolinska Institutet, Stockholm, Sweden; 3 Department of Psychological and Brain Sciences, Indiana University, Bloomington, IN, USA; 4 School of Medical Sciences, Örebro University, Örebro, Sweden; 5 Department of Clinical Sciences, Lund University, Lund, Sweden

**Keywords:** matched cohort study, mortality, prevention, social anxiety disorder, social phobia

## Abstract

**Aims:**

Social anxiety disorder (SAD) is one of the most common anxiety disorders and is associated with significant impairment and societal costs. The association between SAD and mortality remains poorly understood, partly because in epidemiological research it is rarely studied independently from other anxiety disorders. In this population-based matched cohort and sibling control study, we estimated the risk of all-cause and cause-specific mortality in individuals with SAD.

**Methods:**

From a population of individuals born from 1932 and living in Sweden between 1997 and 2020, we identified all cases of SAD (Swedish ICD-10 code F40.1) in the National Patient Register. Each of these individuals was matched on sex, birth year and county of residence with 10 individuals who had never received a diagnosis. Mortality data were extracted from the Cause of Death Register. Risks were estimated using Cox proportional hazards regression models. Models adjusted for sociodemographic covariates and other lifetime psychiatric disorders. We also identified all clusters of full siblings and conducted within-sibling comparisons to account for unmeasured familial confounding.

**Results:**

The matched cohort included 57,360 individuals with SAD and 573,600 unexposed individuals. During the follow-up, 2355 deaths were registered within the exposed cohort vs. 7800 deaths in the matched cohort (crude mortality rates, 5.25 and 1.73 per 1000 person-years, respectively). The full cohort was followed up for a mean of 7.87 years (standard deviation 5.23). In models adjusting for sociodemographic variables, individuals with SAD had a 2.24-fold increased hazard of all-cause mortality (95% confidence interval [CI], 2.13–2.35). The increased risk was observed for both natural (adjusted hazard ratio [HR], 1.62; 95% CI 1.52–1.72) and unnatural causes of death (HR, 4.18; 95% CI 3.82–4.58). The results were robust to additional adjustment for psychiatric comorbidities, but the magnitude of the associations was attenuated, particularly when adjusting for substance use disorders. In the sibling cohort, 39,993 individuals with SAD were compared with their 64,640 unaffected siblings. While the estimates were also attenuated, they remained statistically significant (HR for all-cause mortality, 1.40; 95% CI 1.36–1.45).

**Conclusions:**

Individuals with SAD face an increased risk of mortality, attributable primarily to unnatural causes of death, such as suicide, but also to natural causes, even after adjusting for socioeconomic variables. Psychiatric comorbidities, particularly substance use disorders, and shared familial factors may also contribute to this excess death. Further study of underlying mechanisms may inform prevention and early intervention strategies to reduce mortality in this vulnerable population.

## Introduction

Social anxiety disorder (SAD), also known as social phobia, is an early-onset mental disorder characterised by strong fear of social situations in which the individual may be negatively evaluated by others (American Psychiatric Association, 2013). SAD is one of the most common anxiety disorders, with a lifetime prevalence of around 12% (Ruscio *et al.*, 2008), and is associated with significant impairment and societal costs (Patel *et al.*, 2002; Stein *et al.*, 2017; Vilaplana-Pérez *et al.*, 2021).

Epidemiological studies suggest an increased risk of mortality due to both natural and unnatural causes of death in individuals with anxiety disorders (Meier *et al.*, 2016; Plana-Ripoll *et al.*, 2019; Weye *et al.*, 2020; Russell *et al.*, 2023). Fewer studies have focused specifically on mortality in SAD, and results have been mixed. A US-based study using data from the Epidemiologic Catchment Area Program showed no significantly increased risk of death in SAD (adjusted proportional hazard ratio [HR], 1.08; 95% confidence interval [CI] 0.89–1.32) when comparing 324 individuals with the disorder (105 deceased during the study period) with people without the disorder (Eaton *et al.*, 2013). Similarly, in a German study including 77 individuals with SAD (10 deceased), their time to death was not significantly shorter, compared to the general population (adjusted HR, 1.29; 95% CI 0.68–2.42) (John *et al.*, 2020). In contrast, a Danish register-based study reported an adjusted mortality rate ratio of 1.50 (95% CI 1.20–1.84) for all causes of death, 1.51 (95% CI 1.21–1.85) for natural causes, and 2.19 (95% CI 1.70–2.77) for unnatural causes in individuals with SAD, compared with the general population (Meier *et al.*, 2016). However, deaths due to specific causes within these broader categories were not reported. Because SAD is associated with certain risk behaviours (e.g., alcohol misuse, cannabis dependence) (Buckner *et al.*, 2008; Rosenström and Torvik, 2023), which are in turn associated with various somatic conditions, a more detailed understanding of the causes that contribute to the excess mortality due to natural causes in this group is warranted. Additionally, previous research has established an association between SAD and suicidal ideation and suicide attempts (Thibodeau *et al.*, 2013; Buckner *et al.*, 2017; Leigh *et al.*, 2023), but studies focusing on suicide deaths are rare. One Taiwanese study reported a 3-fold increased risk of suicide in a cohort of over 15,000 individuals with SAD, compared with population controls (Wei *et al.*, 2025), but studies in other populations and contexts are lacking.

In this population-based matched cohort and sibling cohort study, we estimated the risk of all-cause and cause-specific mortality in over 57,000 individuals with SAD, compared with a cohort of demographically matched unexposed individuals. Models adjusted for sociodemographic covariates and other psychiatric disorders. We hypothesised that adjusting for other psychiatric disorders would attenuate, but not eliminate, the magnitude of the associations. To further adjust for unmeasured familial confounding, we employed a sibling control design comparing SAD-exposed individuals with their unexposed full siblings.

## Methods

The Swedish Ethical Review Authority approved the study. Informed consent for register-based studies is waved because participants are not identifiable. The reporting follows the Strengthening the Reporting of Observational Studies in Epidemiology (STROBE) guidelines.

### Data sources

Several Swedish population-based registers were linked through the unique identification number assigned to each Swedish citizen (Ludvigsson *et al.*, 2009), including: (1) the National Patient Register (NPR), containing diagnostic information based on the International Classification of Diseases (ICD), with coverage for inpatient psychiatric diagnoses from 1973 and for outpatient consultations in specialised care from 2001 (Ludvigsson *et al.*, 2011); (2) the Cause of Death Register, including dates of death and codes for underlying and contributory causes of death based on ICD codes for all deaths of Swedish citizens, since 1952 (Brooke *et al.*, 2017); (3) the Census register, containing data from individuals living in the country since 1960; (4) the Total Population Register, containing data on all Swedish inhabitants since 1968 (Ludvigsson *et al.*, 2016); (5) the Migration Register, containing data on immigrations and emigrations (Ludvigsson *et al.*, 2016); (6) the Longitudinal Integration Database for Health Insurance and Labor Studies, containing sociodemographic information from all individuals living in Sweden since 1990 (Ludvigsson *et al.*, 2019); and (7) the Multi-Generation Register, which connects every person born in Sweden since 1932 and ever registered as living in the country after 1960 to their parents (Ekbom, 2011).

### Study population

We included all individuals born from 1932 that were at least 6 years of age and living in Sweden at any time between 1 January 1997 and 31 December 2020. Individuals that had emigrated or died before 1997 or age 6 years were excluded.

We used a matched cohort to estimate the risk of all-cause and cause-specific death in individuals with SAD (exposed), compared to individuals without SAD (unexposed). Each exposed individual was matched on sex, birth year and county of residence at the time of the first recorded SAD diagnosis with 10 individuals who had never received a SAD diagnosis by the date of diagnosis of the corresponding exposed individual. For exposed individuals, the cohort entry date was the date of the first registered SAD diagnosis. Unexposed individuals were assigned the same cohort entry date as their matched exposed counterparts.

Additionally, from the study population, we identified all clusters of full siblings (i.e., those sharing the same biological mother and father), regardless of their SAD exposure, and assigned them a family identification number. In this cohort, the cohort entry date was the date of their sixth birthday, 1 January 1997, or date of immigration, whichever came last.

### Exposure

Individuals were considered exposed if they had a diagnosis of SAD in the NPR (Swedish ICD-10 code F40.1) recorded from 1 January 1997 to 31 December 2020 (i.e., end of the study period), if diagnosed at the age of 6 years or older, to avoid potential misclassifications (Vilaplana-Pérez *et al.*, 2021). This code has shown good validity (positive predictive value = 0.81) and substantial inter-rater reliability (κ = 0.72) (Vilaplana-Pérez *et al.*, 2020).

### Outcomes

All-cause mortality and specific underlying causes of death for individuals that died during the study period were extracted from the Cause of Death Register. Specific causes of death followed the ICD-10 chapters (Supplementary Table 1), and were grouped into natural (all causes except those classified under the ‘external causes of morbidity and mortality’ group) and unnatural (those classified as ‘external causes of morbidity and mortality’). Any causes of death with <10 individual deaths in either the exposed or unexposed cohorts and those classified as ‘codes for special purposes’ in the ICD were grouped under ‘other causes of death’.

### Covariates

Sociodemographic information included country of birth (Sweden or outside of Sweden), level of education (elementary [≤9 years], secondary [10–12 years] or higher education [>12 years]), civil status (single, divorced or widowed, or married or cohabiting), and family income level (lowest 20%, middle or top 20%). The first registered record after the diagnosis of SAD was used for analysis. Lifetime psychiatric disorders were retrieved from the NPR and grouped into: (1) neurodevelopmental disorders; (2) psychotic disorders; (3) bipolar disorders; (4) depressive disorders; (5) phobic, anxiety, obsessive–compulsive and stress disorders (except for SAD), hereafter other anxiety disorders; (6) eating disorders; and (6) substance use disorders (Supplementary Table 2).

### Statistical analyses

Mortality rates per 1000 person-years were calculated for both the exposed and unexposed cohorts. Survival curves were calculated using Kaplan–Meier survival estimates.

In the matched cohort, we used stratified Cox proportional hazards regression analyses, stratified on matching clusters, to estimate HRs with 95% CIs for time to death in individuals with SAD, compared with matched unexposed individuals. Individuals were followed from the cohort entry date until the date of death (outcome), date of emigration from Sweden or the end of the study period, whichever occurred first. Unexposed individuals were additionally censored at the date they changed their exposure status, if they were diagnosed with SAD after the cohort entry date. Time since the cohort entry date was used as the underlying time scale. The analysis was first conducted for all causes of mortality and then separately for each specific cause; for the cause-specific analyses, we used cause-specific hazards and thus censored individuals at time of death due to a different cause than the one analysed. Model 1 adjusted for the matching variables (i.e., birth year, sex and county of residence) at the time of the first SAD diagnosis (and at the corresponding date for matched unexposed individuals). Model 2 further controlled for country of birth and sociodemographic variables (i.e., education, civil status and income). Missing data on these covariates were marked as unknown and then included in the Cox models as nominal variables. We also estimated the associations from Models 1 and 2 separately by sex. We further adjusted Model 2 for different groups of psychiatric comorbidities, one at a time (Model 3).

In the sibling cohort, we compared individuals with SAD with their unexposed full siblings on the same mortality outcomes. Using time-varying exposures, which allowed for siblings to change exposure status, each sibling was followed from the date of their sixth birthday, 1 January 1997, or date of immigration, whichever occurred last, until the date of death, emigration from Sweden or the end of the study period, whichever occurred first. Stratified Cox proportional hazards regression models, stratified on each sibling cluster and using attained age as the underlying time scale, were adjusted for sex and birth year for each sibling (Model 1) and then, additionally, for sociodemographic variables (Model 2).

Data management and analyses were performed using SAS 9.4 (SAS Institute Inc.) and Stata 18.0 (StataCorp LLC). All tests employed two-tailed significance set at p < 0.05.

## Results

### Matched cohort

**Figure 1.**
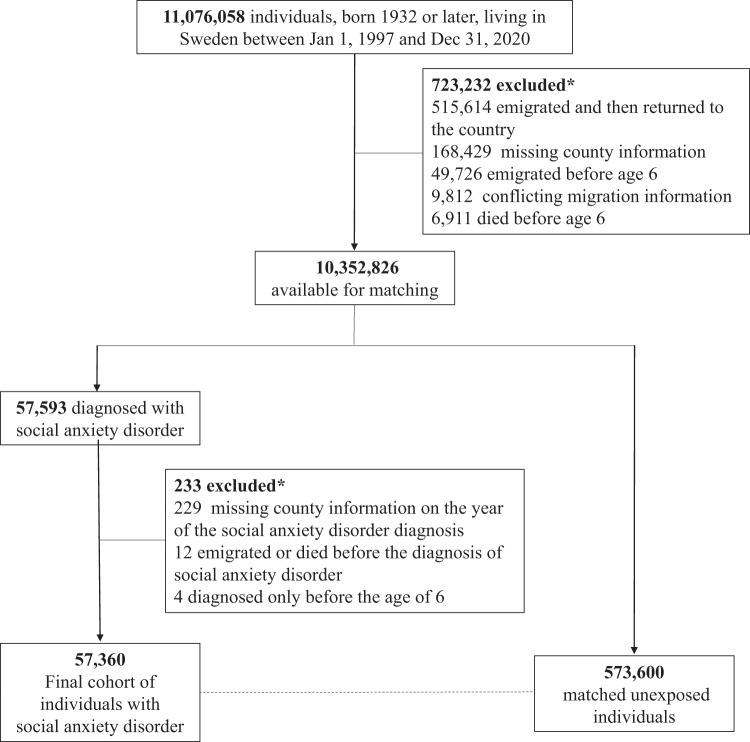
Flow of study participants.

We first identified 11,076,058 individuals who were born in 1932 or later and lived in Sweden at some point between 1 January 1997 and 31 December 2020. After applying several inclusion criteria (see flow of participants in Fig. 1), we identified 57,360 individuals who constituted the final exposed cohort and were matched with 10 individuals without SAD on sex, birth year and county. The full cohort was followed up for a mean of 7.87 years (standard deviation 5.23).

*Numbers add up to more than the total of excluded because individuals could be excluded for more than one reason.

For those exposed, the median age at first diagnosis of SAD was 27.56 years (interquartile range, 20.62–39.12). Individuals with SAD were significantly more likely than matched individuals to be born in Sweden, have a lower level of education, be single, divorced or widowed, have a lower family income, and have higher rates of other psychiatric disorders (Table 1).

**Table 1.** Baseline characteristics and follow-up data of study participants

	Matched cohort		Sibling cohort^b^	
Characteristics^a^	Individuals with social anxiety disorder (*N* = 57,360)	Matched unexposed individuals (*N* = 573,600)	Individuals with social anxiety disorder (*N* = 39,933)	Unaffected full siblings (*N* = 64,640)
**Birth year**				
Before 1960	5521 (9.63)	55,210 (9.63)	3673 (9.20)	8668 (13.41)
1960–1969	7313 (12.75)	73,130 (12.75)	4987 (12.49)	8635 (13.36)
1970–1979	9327 (16.26)	93,270 (16.26)	6127 (15.34)	10,397 (16.08)
1981–1989	14,847 (25.88)	148,470 (25.88)	10,479 (26.24)	15,866 (24.55)
1991–1999	14,985 (26.12)	149,850 (26.12)	10,772 (26.98)	14,788 (22.88)
2000–2009	5347 (9.32)	53,470 (9.32)	3881 (9.72)	5795 (8.97)
After 2009	20 (0.03)	200 (0.03)	14 (0.04)	491 (0.76)
**Sex**, *n* (%)				
Female	32,493 (56.65)	324,930 (56.65)	22,466 (56.26)	31,409 (48.59)
Male	24,867 (43.35)	248,670 (43.35)	17,467 (43.74)	33,231 (51.41)
**Country of birth**, *n* (%)				
Sweden	52,253 (91.10)	464,339 (80.95)	38,648 (96.78)	61,955 (95.85)
Abroad	5,107 (8.90)	109,261 (19.05)	1,285 (3.22)	2,685 (4.15)
**Educational level**, *n* (%), years				
Elementary education	15,620 (27.23)	80,423 (14.02)	10,596 (26.53)	11,737 (18.16)
Secondary education	25,329 (44.16)	242,513 (42.28)	17,756 (44.46)	29,076 (44.98)
Higher education	15,234 (26.56)	232,764 (40.58)	10,756 (26.94)	20,998 (32.48)
Unknown	1177 (2.05)	17,900 (3.12)	825 (2.07)	2829 (4.38)
**Civil status**, *n* (%)				
Single, divorced or widowed	47,999 (83.68)	386,213 (67.33)	33,580 (84.01)	45,056 (69.70)
Married or cohabiting	8843 (15.42)	182,188 (31.76)	5970 (14.95)	17,640 (27.29)
Unknown	518 (0.90)	5199 (0.91)	383 (0.96)	1944 (3.01)
**Family income level**, *n* (%)				
Lowest 20%	24,950 (43.50)	109,166 (19.03)	17,920 (44.88)	16,348 (25.29)
Middle	26,307 (45.86)	340,709 (59.40)	17,723 (44.38)	36,884 (57.06)
Top 20%	5559 (9.69)	115,969 (20.22)	3907 (9.78)	9458 (14.63)
Unknown	544 (0.95)	7756 (1.35)	383 (0.96)	1950 (3.02)
**Any lifetime psychiatric disorders**, *n* (%)	51,966 (90.60)	106,572 (18.58)	35,981 (90.10)	19,094 (29.54)
Neurodevelopmental disorders	19,667 (34.29)	31,311 (5.46)	13,692 (34.29)	6858 (10.61)
Psychotic disorders	3730 (6.50)	6234 (1.09)	2470 (6.19)	1285 (1.99)
Bipolar disorders	6475 (11.29)	7748 (1.35)	4549 (11.39)	1575 (2.44)
Depressive disorders	38,036 (66.31)	46,505 (8.11)	26,329 (65.93)	8894 (13.76)
Other anxiety disorders	34,836 (60.73)	44,482 (7.75)	23,695 (59.34)	7749 (11.99)
Eating disorders	4863 (8.48)	10,446 (1.82)	3494 (8.75)	1478 (2.29)
Substance use disorders	15,521 (27.06)	31,606 (5.51)	10,427 (26.11)	6226 (9.63)
**Age at death**, median (IQR), years	50.95 (36.13−62.34)	60.23 (48.26−68.80)	50.27 (35.79−61.42)	55.54 (41.68−66.42)
**Follow-up time**, mean (SD), years^c^	7.82 (5.17)	7.88 (5.24)	21.76 (3.96)	21.56 (4.69)

a All differences between individuals with social anxiety disorder and matched unexposed individuals are significant at *P* < 0.0001, except for sex (matching variable) and follow-up time (*P* = 0.005). All differences between individuals with social anxiety disorder and their unaffected siblings are significant at *P* < 0.001, except for mean age at death (*P* = 0.027).

b The table only reports the characteristics of the siblings in the cohort discordant for social anxiety disorder at the end of the follow-up. Note that more siblings may contribute to the analysis.

c In the matched cohort, individuals with social anxiety disorder and the matched unexposed cohort were followed from the cohort entry date (i.e., date of the first diagnosis of social anxiety disorder, and the corresponding date for comparators), while in the sibling cohort, each sibling was followed from their sixth birthday, 1 January 1997 or the date of immigration, whichever occurred last, which explains the difference follow-up time between cohorts.

**Figure 2.**
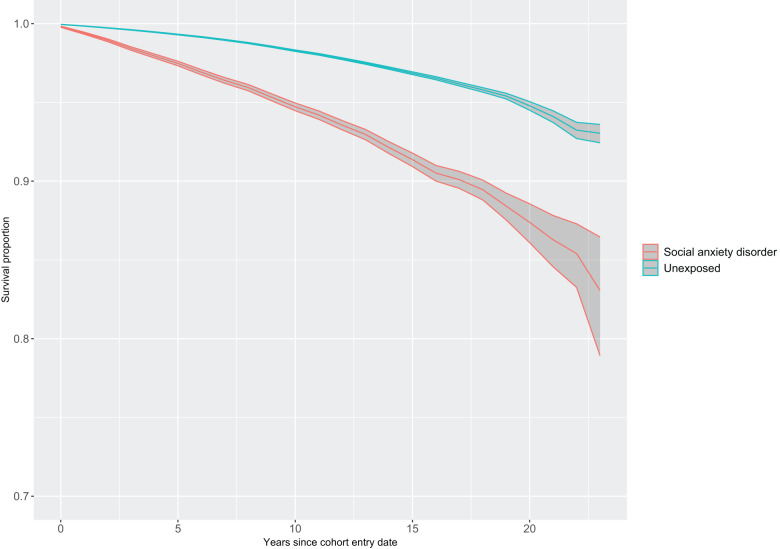
Survival proportion (Kaplan–Meier estimates with 95% confidence intervals) under the assumption of no competing risks for all-cause mortality in individuals with social anxiety disorder and matched unexposed individuals.

During the follow-up, 2355 deaths were registered within the exposed cohort vs. 7800 deaths in the matched cohort. This translated into crude mortality rates of 5.25 and 1.73 per 1000 person-years, respectively. Kaplan–Meier survival curves for both exposed and matched unexposed cohorts are shown in Figure 2. Estimates adjusting for matching variables (Model 1) showed an increased risk of all-cause mortality in individuals with SAD (HR, 3.23; 95% CI 3.08–3.38), compared with unexposed individuals. Risks were also elevated for the exposed cohort for natural (HR, 2.15; 95% CI 2.02–2.28) and unnatural causes of death (HR, 7.44; 95% CI 6.86–8.06), as well as for all the specific causes within these broader groups, except for the category ‘other natural causes of death’ (Table 2). The magnitude of these risks was considerably reduced when the model further adjusted for sociodemographic variables (Model 2; all-cause mortality: HR, 2.24; 95% CI, 2.13–2.35, natural causes of death: HR, 1.62; 95% CI 1.52–1.72, unnatural causes of death: HR, 4.18; 95% CI 3.82–4.58). The risks of death due to certain infectious and parasitic diseases and diseases of the nervous system were no longer statistically significant. However, individuals with SAD still had a significantly increased risk of death due to mental and behavioural disorders (a group that mostly included deaths attributable to the use of alcohol), diseases of the digestive system (e.g., alcoholic liver disease, fibrosis and cirrhosis of the liver), diseases of the genitourinary system (e.g., kidney failure), diseases of the respiratory system (e.g., chronic obstructive pulmonary diseases, pneumonia), symptoms, signs and abnormal clinical laboratory findings (e.g., unknown causes of mortality), diseases of the circulatory system (e.g., chronic ischemic heart diseases, acute myocardial infarction), endocrine, nutritional and metabolic diseases (e.g., diabetes mellitus, obesity), and neoplasms (e.g., neoplasm of bronchus and lung, neoplasm of pancreas), as well as suicides, accidents and other unnatural causes of death (Table 2).

**Table 2.** Hazard ratios (HRs) with 95% confidence intervals (CIs) for all-cause and cause-specific mortality among individuals with social anxiety disorder, compared to matched unexposed individuals

Causes of death	Individuals with social anxiety disorder (*N* = 57,360) *n* (%)	Matched unexposed individuals (*N* = 573,600) *n* (%)	HR (95% CI) Model 1, minimally adjusted^a^	HR (95% CI) Model 2, additionally adjusted for socioeconomic variables^b^
**All-cause mortality**	2355 (4.11)	7800 (1.36)	**3.23 (3.08−3.38)**	**2.24 (2.13−2.35)**
**Natural causes of death**	1308 (2.28)	6395 (1.11)	**2.15 (2.02−2.28)**	**1.62 (1.52−1.72)**
Certain infectious and parasitic diseases	21 (0.04)	116 (0.02)	**1.79 (1.12−2.84)**	1.16 (0.71−1.90)
Neoplasms	371 (0.65)	2808 (0.49)	**1.32 (1.19−1.47)**	**1.19 (1.07−1.34)**
Endocrine, nutritional, and metabolic diseases	53 (0.09)	223 (0.04)	**2.35 (1.75−3.18)**	**1.46 (1.05−2.02)**
Mental and behavioural disorders	66 (0.12)	154 (0.03)	**4.27 (3.20−5.71)**	**2.83 (2.07−3.89)**
Diseases of the nervous system	58 (0.10)	305 (0.05)	**1.91 (1.44−2.52)**	1.35 (1.00−1.83)
Diseases of the circulatory system	393 (0.69)	1,592 (0.28)	**2.52 (2.25−2.81)**	**1.72 (1.53−1.93)**
Diseases of the respiratory system	109 (0.19)	373 (0.07)	**2.92 (2.36−3.62)**	**2.09 (1.66−2.63)**
Diseases of the digestive system	128 (0.22)	325 (0.06)	**3.89 (3.17−4.78)**	**2.34 (1.87−2.92)**
Diseases of the genitourinary system	14 (0.02)	53 (0.01)	**2.62 (1.45−4.71)**	**2.14 (1.14−4.02)**
Symptoms, signs and abnormal clinical and laboratory findings, not elsewhere classified	70 (0.12)	241 (0.04)	**2.87 (2.20−3.74)**	**1.87 (1.39−2.51)**
Other natural causes of death^c^	25 (0.04)	205 (0.04)	1.21 (0.80−1.83)	0.76 (0.49−1.18)
**Unnatural causes of death**	1047 (1.83)	1405 (0.24)	**7.44 (6.86−8.06)**	**4.18 (3.82−4.58)**
Accidents	371 (0.65)	581 (0.10)	**6.30 (5.53−7.17)**	**3.27 (2.82−3.80)**
Suicides	655 (1.14)	759 (0.13)	**8.60 (7.74−9.55)**	**5.00 (4.44−5.62)**
Other unnatural causes of death	21 (0.04)	65 (0.01)	**3.20 (1.96−5.24)**	**2.22 (1.29−3.85)**

*Note*: Significant estimates are highlighted in bold.

a Adjusted for all matching variables (i.e., sex, birth year, county of residence at the time of the social anxiety disorder diagnosis).

b Adjusted for all matching variables, country of birth (Sweden *vs.* abroad) and latest recorded highest level of education, family income level and civil status.

c Includes all groups with a small number of deaths (≤10) and the causes of death classified in the ICD as ‘codes for special purposes’.

Both females and males with SAD, compared with matched unexposed counterparts, had similar risks of death (Supplementary Tables 3 and 4). However, the risk estimates for some of the natural causes of death in individuals with SAD differed according to sex, as indicated by non-overlapping CIs when comparing the estimates for females and males. The risk of death due to mental and behavioural disorders was associated with SAD in males but not in females, while the risk of death due to neoplasms and endocrine, nutritional and metabolic diseases was associated with SAD in females, but not males.

Analyses additionally adjusting for other psychiatric disorders (Model 3; Table 3) showed attenuations of the magnitude of the risks to a different degree, with the estimates for all-cause mortality ranging from an HR of 1.46 (95% CI, 1.39–1.55) when adjusting for substance use disorders to an HR of 2.21 (95% CI, 1.60–2.06) when adjusting for eating disorders. Substance use disorders had the largest impact on the risk estimates. When adjusting for this group, the risks of all-cause mortality, natural causes of death, unnatural causes of death and the specific groups of diseases of the circulatory system, suicides and accidents were significantly reduced (non-overlapping CIs with the corresponding HRs from Model 2 in the main analysis), albeit they were still significant. Additionally, the risk of death due to diseases of the digestive system attenuated to the null when further adjusting for substance use disorders. Adjustment for depressive and other anxiety disorders also significantly reduced the magnitude of the estimates for all-cause mortality, unnatural causes of death and suicide. Adjustment for depressive disorders significantly reduced the risk of natural causes of death, while adjustment for other anxiety disorders significantly reduced the risk of accidents, although they were still significantly elevated.

**Table 3.** Hazard ratios (HRs) with 95% confidence intervals (CIs) for all-cause and cause-specific mortality among individuals with social anxiety disorder, compared to matched unexposed individuals, further adjusted for different groups of psychiatric comorbidities (one group at a time)

Causes of death	HR (95% CI) Model 3, additionally adjusted for neuro-developmental disorders^a^	HR (95% CI) Model 3, additionally adjusted for psychotic disorders^a^	HR (95% CI) Model 3, additionally adjusted for bipolar disorders^a^	HR (95% CI) Model 3, additionally adjusted for depressive disorders^a^	HR (95% CI) Model 3, additionally adjusted for other anxiety disorders^a^	HR (95% CI) Model 3, additionally adjusted for eating disorders^a^	HR (95% CI) Model 3, additionally adjusted for substance use disorders^a^
**All-cause mortality**	**2.13 (2.02−2.24)**	**2.13 (2.03−2.24)**	**2.11 (2.01−2.22)**	**1.64 (1.54−1.74)**	**1.76 (1.66−1.87)**	**2.21 (2.10−2.33)**	**1.46 (1.39−1.55)**
**Natural causes of death**	**1.61 (1.51−1.72)**	**1.54 (1.44−1.64)**	**1.56 (1.46−1.67)**	**1.33 (1.23−1.43)**	**1.44 (1.33−1.55)**	**1.61 (1.51−1.72)**	**1.15 (1.07−1.23)**
Certain infectious and parasitic diseases	1.14 (0.69−1.89)	1.14 (0.69−1.87)	1.12 (0.68−1.86)	0.95 (0.55−1.63)	0.88 (0.49−1.58)	1.16 (0.70−1.90)	0.73 (0.42−1.24)
Neoplasms	**1.20 (1.07−1.35)**	**1.18 (1.05−1.32)**	**1.19 (1.06−1.33)**	1.12 (0.98−1.28)	1.15 (1.00−1.31)	**1.19 (1.07−1.34)**	1.04 (0.92−1.17)
Endocrine, nutritional, and metabolic diseases	**1.48 (1.06−2.08)**	**1.40 (1.01−1.94)**	1.38 (0.99−1.93)	1.00 (0.69−1.43)	**1.57 (1.07−2.31)**	**1.40 (1.01−1.95)**	1.04 (0.73−1.48)
Mental and behavioural disorders	**2.90 (2.10−4.00)**	**2.61 (1.89−3.61)**	**2.71 (1.96−3.74)**	**2.34 (1.63−3.35)**	**2.45 (1.68−3.58)**	**2.77 (2.02−3.81)**	**1.46 (1.02−2.10)**
Diseases of the nervous system	1.32 (0.97−1.80)	1.33 (0.98−1.80)	1.34 (0.99−1.83)	1.03 (0.72−1.46)	**1.44 (1.01−2.05)**	**1.38 (1.02−1.87)**	1.15 (0.83−1.59)
Diseases of the circulatory system	**1.72 (1.52−1.94)**	**1.62 (1.44−1.83)**	**1.65 (1.46−1.86)**	**1.37 (1.19−1.57)**	**1.48 (1.28−1.71)**	**1.72 (1.53−1.94)**	**1.26 (1.11−1.43)**
Diseases of the respiratory system	**2.03 (1.60−2.56)**	**2.05 (1.62−2.59)**	**1.96 (1.55−2.47)**	**1.51 (1.14−1.99)**	**1.51 (1.15−1.99)**	**2.06 (1.64−2.60)**	**1.40 (1.09−1.80)**
Diseases of the digestive system	**2.30 (1.83−2.88)**	**2.25 (1.80−2.82)**	**2.25 (1.79−2.82)**	**2.01 (1.55−2.62)**	**1.70 (1.30−2.23)**	**2.34 (1.87−2.92)**	1.07 (0.82−1.40)
Diseases of the genitourinary system	**2.40 (1.26−4.54)**	**2.15 (1.15−4.03)**	**2.00 (1.05−3.79)**	1.01 (0.45−2.27)	1.73 (0.79−3.78)	**2.09 (1.11−3.93)**	1.82 (0.95−3.50)
Symptoms, signs and abnormal clinical and laboratory findings, not elsewhere classified	**1.74 (1.28−2.36)**	**1.79 (1.32−2.42)**	**1.81 (1.34−2.45)**	**1.46 (1.05−2.03)**	**1.54 (1.10−2.17)**	**1.88 (1.39−2.53)**	1.24 (0.89−1.71)
Other natural causes of death^b^	0.75 (0.48−1.18)	0.75 (0.48−1.18)	0.74 (0.47−1.16)	0.60 (0.35−1.02)	0.64 (0.38−1.09)	0.72 (0.46−1.13)	0.69 (0.43−1.11)
**Unnatural causes of death**	**3.82 (3.48−4.20)**	**4.00 (3.66−4.39)**	**3.81 (3.48−4.18)**	**2.44 (2.20−2.71)**	**2.69 (2.42−2.99)**	**4.09 (3.73−4.48)**	**2.47 (2.23−2.73)**
Accidents	**2.95 (2.52−3.45)**	**3.17 (2.73−3.68)**	**3.07 (2.64−3.58)**	**2.42 (2.03−2.89)**	**2.27 (1.90−2.71)**	**3.26 (2.80−3.79)**	**1.67 (1.40−1.98)**
Suicides	**4.57 (4.05−5.17)**	**4.79 (4.25−5.39)**	**4.49 (3.98−5.06)**	**2.52 (2.21−2.89)**	**3.04 (2.65−3.48)**	**4.85 (4.31−5.46)**	**3.16 (2.77−3.60)**
Other unnatural causes of death	**2.23 (1.27−3.92)**	**2.18 (1.25−3.80)**	**2.09 (1.20−3.64)**	**2.10 (1.13−3.93)**	**2.08 (1.10−3.94)**	**2.26 (1.30−3.90)**	1.71 (0.96−3.04)

*Note*: Significant estimates are highlighted in bold.

a Adjusted for all matching variables (i.e., sex, birth year, county of residence at the time of the social anxiety disorder diagnosis), country of birth (Sweden *vs.* abroad), latest recorded highest level of education, family income level and civil status, and the specified group of psychiatric comorbidities.

b Includes all groups with a small number of deaths (≤10) and the causes of death classified in the ICD as ‘codes for special purposes’.

### Sibling cohort

By the end of the follow-up, among a cohort of 6,363,292 siblings (2,570,996 clusters) from the whole study population, we identified 39,993 who had received a SAD diagnosis and their 64,640 unaffected siblings (Table 1). Individuals with SAD, compared to their unaffected full siblings, were more likely to be born in more recent birth cohorts, male, born in Sweden, less educated, single, divorced or widowed, have a lower family income, and have a significantly higher rate of comorbid psychiatric disorders (Table 1). Individuals with SAD had a higher risk of mortality than their siblings without the diagnosis, although the magnitude of this association was substantially lower than in the matched cohort comparison (HR for all-cause mortality, 1.40; 95% CI 1.36–1.45; HR for natural causes of death, 1.22; 95% CI, 1.17–1.27; HR for unnatural causes of death, 1.71; 95% CI 1.62–1.80). Among the deaths due to specific groups of diseases, results also remained overall robust, albeit the estimates were also reduced. Only the risk of death due to neoplasms became non-significant (HR, 1.01; 95% CI 0.94–1.08), as did the risks of death due to ‘other natural’ and ‘other unnatural’ causes (HR, 0.85; 95% CI 0.68–1.06 and HR, 1.20; 95% CI 0.89–1.62, respectively) (Table 4).

**Table 4.** Hazard ratios (HRs) with 95% confidence intervals (CIs) for all-cause and cause-specific mortality among individuals with social anxiety disorder, compared to their unaffected full siblings

	Individuals with social anxiety disorder (*N* = 39,933)^a^	Unaffected full siblings (*N* = 64,640)^a^	HR (95% CI) Model 1, minimally adjusted^b^	HR (95% CI) Model 2, additionally adjusted for socioeconomic variables^c^
Causes of death	*n* (%)	*n* (%)		
**All-cause mortality**	1541 (3.86)	2320 (3.59)	**1.38 (1.34−1.43)**	**1.40 (1.36−1.45)**
**Natural causes of death**	816 (2.04)	1712 (2.65)	**1.20 (1.15−1.25)**	**1.22 (1.17−1.27)**
Certain infectious and parasitic diseases	16 (0.04)	38 (0.06)	1.07 (0.78−1.45)	1.10 (0.81−1.49)
Neoplasms	235 (0.59)	730 (1.13)	0.97 (0.91−1.04)	1.01 (0.94−1.08)
Endocrine, nutritional, and metabolic diseases	36 (0.09)	51 (0.08)	**1.54 (1.27−1.87)**	**1.54 (1.27−1.87)**
Mental and behavioural disorders	44 (0.11)	71 (0.11)	**1.45 (1.20−1.75)**	**1.41 (1.16−1.73)**
Diseases of the nervous system	37 (0.09)	75 (0.12)	1.12 (0.92−1.35)	1.13 (0.94−1.37)
Diseases of the circulatory system	241 (0.60)	435 (0.67)	**1.33 (1.22−1.44)**	**1.33 (1.23−1.44)**
Diseases of the respiratory system	61 (0.15)	96 (0.15)	**1.33 (1.14−1.54)**	**1.31 (1.13−1.52)**
Diseases of the digestive system	88 (0.22)	99 (0.15)	**1.55 (1.36−1.75)**	**1.51 (1.34−1.72)**
Symptoms, signs and abnormal clinical and laboratory findings, not elsewhere classified	38 (0.10)	45 (0.07)	**1.45 (1.18−1.77)**	**1.48 (1.21−1.82)**
Other natural causes of death^d^	214 (0.54)	403 (0.62)	0.81 (0.65−1.02)	0.85 (0.68−1.06)
**Unnatural causes of death**	725 (1.82)	608 (0.94)	**1.70 (1.61−1.79)**	**1.71 (1.62−1.80)**
Accidents	263 (0.66)	242 (0.37)	**1.63 (1.50−1.77)**	**1.66 (1.52−1.80)**
Suicides	449 (1.12)	337 (0.52)	**1.73 (1.61−1.85)**	**1.73 (1.62−1.85)**
Other unnatural causes of death	13 (0.03)	29 (0.04)	1.21 (0.89−1.63)	1.20 (0.89−1.62)

*Note*: Significant estimates are highlighted in bold.

a The reported counts and frequencies refer to the siblings in the cohort discordant for social anxiety disorder at the end of the follow-up. Note that more siblings may contribute to the analysis.

b Adjusted for sex and birth year for each sibling and sibling order.

c Adjusted for all variables in Model 1 plus country of birth and latest recorded highest level of education, family income level and civil status.

d The adjusted model could not be fitted for this cause of death due to complete separation, which resulted from the low number of observations in certain covariates.^e^Includes all groups with a small number of deaths (≤10) and the causes of death classified in the ICD as ‘codes for special purposes’.

## Discussion

We investigated all-cause and cause-specific mortality in over 50,000 individuals with SAD diagnosed in specialist services, compared with demographically matched unexposed individuals. The risk of mortality in those with SAD was over twofold compared with the unexposed group, attributable primarily to unnatural causes of death, such as suicide, but also to natural causes, even after adjusting for socioeconomic variables.

Our all-cause mortality estimates were higher than those reported in a smaller study in Denmark (Meier *et al.*, 2016), which showed a 50% increased risk of death in individuals with SAD, compared with the general population. Both our and the Danish results (Meier *et al.*, 2016) differed from previous reports that did not find an excess mortality risk in this group (Eaton *et al.*, 2013; John *et al.*, 2020). Methodological differences, with the former studies including data from hospital records and the latter based on data from random samples from the population, may be responsible for the different outcomes. In fact, a systematic review and meta-analysis showed no evidence of increased mortality risk among anxious participants from community samples (Miloyan *et al.*, 2016). This indicates that the risk may only be present in individuals whose symptoms are severe or complex enough to seek treatment and require specialist treatment.

For the first time, we were able to identify specific natural causes of death in individuals with SAD. Deaths due to mental and behavioural disorders, mostly due to use of alcohol, showed the highest risk estimates, followed by deaths related to the digestive, genitourinary, respiratory, circulatory and endocrine systems and neoplasms. Notably, many of these causes are associated with behavioural factors, such as the use of alcohol and other unhealthy lifestyles. This finding is compatible with the so-called self-medication hypothesis by which individuals with SAD consume anxiolytic substances, such as alcohol, in order to temporarily alleviate social anxiety symptoms (Carrigan and Randall, 2003; Rosenström and Torvik, 2023).

There were significant sex differences in the risks of some natural causes of death. Namely, there was no association between SAD and deaths due to mental disorders in females, while there was a significant association between SAD and this cause of death in males, which may have been driven by a higher prevalence of use of substances in males. On the other hand, females with SAD had a higher risk of death due to neoplasms and endocrine, nutritional and metabolic diseases, while risks of death due to these causes were not significant in males with SAD. The reasons for these differences are unknown and puzzling given that females are known to have higher rates of healthcare-seeking behaviours across all health concerns (Rata Mohan *et al.*, 2025). Further research should explore potential putative mechanisms behind these differences, including sex differences in stress-response, inflammatory, cardiovascular and other biological variables, comorbid somatic illness and substance use, and help-seeking and treatment adherence behaviours.

Our results on deaths by suicide are in line with those from a register-based Taiwanese study (Wei *et al.*, 2025). To our knowledge, the finding that SAD is associated with an increased risk of death due to accidents is novel and warrants further exploration. Again, it could be hypothesised that the excess number of accidents in this group is associated with the use of alcohol and other substances, as well as with the relatively high levels of neurodevelopmental disorder comorbidity in our cohort. Specifically, previous research has shown that attention-deficit/hyperactivity disorder is associated with transport accidents (Chang *et al.*, 2014, 2017; Mataix‐Cols *et al.*, 2021).

Nonetheless, additional adjustment of our risk estimates for psychiatric comorbidities, especially substance use disorders, but also depressive and other anxiety disorders, considerably attenuated the mortality estimates, indicating that these comorbidities are likely to explain some of the risk, but did not fully account for it. The sibling comparison also showed an attenuation of the risk of death. This suggests that shared environmental and genetic factors may also contribute, at least to some extent, to the observed association between SAD and mortality.

Importantly, like in other disorders in the broad anxiety spectrum, such as obsessive-compulsive disorder (Fernández de la Cruz *et al.*, 2024), the majority of excess deaths in the SAD cohort in our study are potentially amenable to prevention or early interventions. Those caring for individuals with SAD should put specific emphasis on the management of comorbid psychiatric conditions (specifically substance use disorders, depression and other anxiety disorders) and the long-term monitoring of suicide risk and somatic health, particularly in individuals with substance use.

### Strengths and limitations

To our knowledge, this was the first study systematically investigating specific causes of death in a large cohort of individuals with SAD. The ICD-10 SAD codes are well validated in the NPR (Vilaplana-Pérez *et al.*, 2020). Additionally, the Swedish Cause of Death Register has an outstanding nationwide coverage (Brooke *et al.*, 2017). We also adjusted for relevant socioeconomic factors and lifetime psychiatric comorbidities. An additional sibling comparison allowed us to consider unmeasured familial confounding.

The study also has limitations. First, the NPR only includes diagnoses in specialist services, with information from outpatient care only available from 2001, which may have resulted in the inclusion of individuals with SAD in the more severe end of the spectrum, given that many anxiety patients are generally managed in primary care. Nonetheless, our previous validation of the SAD codes in the NPR showed a fairly normal distribution of cases according to their severity (Vilaplana-Pérez *et al.*, 2020). Second, because SAD as an independent diagnosis within the broad neurotic disorders category only appears in the ICD-10, implemented in Sweden in 1997, our exposed cohort only includes individuals with a diagnosis following that date. This may have led to a potential misclassification of cases (e.g., if individuals were diagnosed for the first time as socially anxious before 1997 and never diagnosed again). Also, our cohort was relatively young (note that the mean age at death for the non-exposed matched cohort is around 60, significantly younger than the life expectancy in Sweden), and it may have been too early for many individuals to die from causes that tend to appear later in life. Third, some of the covariates in our adjusted models could be mediators rather than confounders, potentially underestimating the magnitude of the true associations. However, we also presented a model that did not adjust for these variables. Although this model showed higher estimates, the additionally adjusted analyses still showed significant risks for the outcomes of interest. Fourth, our study did not analyse additional variables that may be relevant for mortality, such as the severity of SAD, childhood experiences, lifestyle factors, use of medication or healthcare utilisation. Finally, our study was conducted in a country with universal healthcare and it is unknown whether results generalise to countries with different models.

## Conclusion

Individuals with SAD face an increased risk of mortality, particularly due to unnatural causes of death, such as suicide. Psychiatric comorbidities, specifically substance use disorders, significantly contribute to this excess death. Clarifying the underlying mechanisms may inform targeted prevention strategies and ultimately reduce mortality in this population.

## Supporting information

10.1017/S2045796026100535.sm001 Fernández de la Cruz et al. supplementary material 1 Fernández de la Cruz et al. supplementary material

10.1017/S2045796026100535.sm002 Fernández de la Cruz et al. supplementary material 2 Fernández de la Cruz et al. supplementary material

## Data Availability

Sharing of the individual-level data is restricted by Swedish data protection laws and data underlying the reported findings cannot be deposited in publicly accessible archives. In this study, data were obtained from the National Patient Register held by the Swedish National Board of Health and Welfare (*Socialstyrelsen*; http://www.socialstyrelsen.se/english) and the Total Population Register maintained by Statistics Sweden (*SCB*; http://www.scb.se/en/), among others. For enquiries about access to data, any interested parties can contact the data owners via registerservice@socialstyrelsen.se and information@scb.se.

## Supplementary material

The supplementary material for this article can be found at https://doi.org/10.1017/S2045796026100535.
