# Supplementary material for: All-cause and cause-specific mortality in social anxiety disorder: a matched cohort and sibling cohort study
Source: Epidemiol Psychiatr Sci. 2026 Mar 27;35:e13. doi: 10.1017/S2045796026100535 (PMC13122535; doi:10.1017/S2045796026100535)
Supplement: Fernández de la Cruz et al. supplementary material 1 — Fernández de la Cruz et al. supplementary material [file S2045796026100535sup001.docx]

**STROBE Statement—checklist of items that should be included in reports of observational studies.**

|  | | | | | Item No. | Recommendation | | Page  No. | Relevant text from manuscript | |  |
| --- | --- | --- | --- | --- | --- | --- | --- | --- | --- | --- | --- |
| **Title and abstract** | | | | | 1 | (*a*) Indicate the study’s design with a commonly used term in the title or the abstract | | 1 | *“a matched cohort and sibling cohort study”* | |  |
|  |  |  |  |  |  | (*b*) Provide in the abstract an informative and balanced summary of what was done and what was found | | 2 | Full abstract (page 2). | |  |
| Introduction | | |  |  |  |  |  |  |  |  |  |
| Background/rationale | | | | | 2 | Explain the scientific background and rationale for the investigation being reported | | 3-4 | Full Introduction (pages 3-4). | |  |
| Objectives | | | | | 3 | State specific objectives, including any prespecified hypotheses | | 4 | *“In this population-based matched cohort study with sibling control, we estimated the risk of all-cause and cause-specific mortality in over 57,000 individuals with SAD, compared to a cohort of demographically-matched unexposed individuals. Models adjusted for a range of covariates, including sociodemographic covariates and other psychiatric disorders.”* | |  |
| Methods |  |  |  |  |  |  |  |  |  |  |  |
| Study design | | | | | 4 | Present key elements of study design early in the paper | | 4-5 | *“In this matched cohort and sibling cohort study, several Swedish population-based registers were linked…”* | |  |
| Setting | | | | | 5 | Describe the setting, locations, and relevant dates, including periods of recruitment, exposure, follow-up, and data collection | | 4-6 | Data sources, Exposure, Outcomes, and Covariates sections (pages 4-6). | |  |
| Participants | | | | | 6 | (*a*) *Cohort study*—Give the eligibility criteria, and the sources and methods of selection of participants. Describe methods of follow-up  *Case-control study*—Give the eligibility criteria, and the sources and methods of case ascertainment and control selection. Give the rationale for the choice of cases and controls  *Cross-sectional study*—Give the eligibility criteria, and the sources and methods of selection of participants | | 4-5 | Data sources and study population sections (4-5) and Supplementary Figure 1 (flow of study participants)- | |  |
|  |  |  |  |  |  | (*b*) *Cohort study*—For matched studies, give matching criteria and number of exposed and unexposed  *Case-control study*—For matched studies, give matching criteria and the number of controls per case | | 5, 8 | *“Each exposed individual was matched on sex, birth year, and county of residence at the time of the first recorded SAD diagnosis with 10 individuals who had never received a SAD diagnosis by the date of diagnosis of the corresponding exposed individual.”*  *“The remaining 57,360 individuals constituted the final exposed cohort. Each individual within this cohort was matched with 10 individuals without SAD on sex, birth year, and county.”* | |  |
| Variables | | | | | 7 | Clearly define all outcomes, exposures, predictors, potential confounders, and effect modifiers. Give diagnostic criteria, if applicable | | 5-6 | Exposure, Outcomes, and Covariates sections (pages 5-6). | |  |
| Data sources/ measurement | | | | | 8 | For each variable of interest, give sources of data and details of methods of assessment (measurement). Describe comparability of assessment methods if there is more than one group | | 4-5 | Data sources section (pages 4-5). | |  |
| Bias | | | | | 9 | Describe any efforts to address potential sources of bias | | 6-7 | Covariates and statistical analysis section (pages 6-7). | |  |
| Study size | | | | | 10 | Explain how the study size was arrived at | | 5, 7-8 | Study population section (page 5), matched cohort section (pages 7-8), and Supplementary Figure 1 (flow of study participants). | |  |
| Quantitative variables | | | | | 11 | Explain how quantitative variables were handled in the analyses. If applicable, describe which groupings were chosen and why | | 6-7 | Statistical analysis section (pages 6-7). | |  |
| Statistical methods | | | | | 12 | (*a*) Describe all statistical methods, including those used to control for confounding | | 6-7 | Statistical analysis section (pages 6-7). | |  |
|  |  |  |  |  |  | (*b*) Describe any methods used to examine subgroups and interactions | | 7 | *“We also estimated the associations from Models 1 and 2 separately by sex. We further adjusted Model 2 for different groups of psychiatric comorbidities, one at a time (Model 3).”* | |  |
|  |  |  |  |  |  | (*c*) Explain how missing data were addressed | | 7 | *“Missing data on these covariates were marked as unknown and then included in the Cox models as nominal variables.”* | |  |
|  |  |  |  |  |  | (*d*) *Cohort study*—If applicable, explain how loss to follow-up was addressed  *Case-control study*—If applicable, explain how matching of cases and controls was addressed  *Cross-sectional study*—If applicable, describe analytical methods taking account of sampling strategy | | 5 | *“We used a matched cohort to estimate the risk of all-cause and cause-specific death in individuals with SAD (exposed), compared to individuals without SAD (unexposed). Each exposed individual was matched on sex, birth year, and county of residence at the time of the first recorded SAD diagnosis with 10 individuals who had never received a SAD diagnosis by the date of diagnosis of the corresponding exposed individual. For exposed individuals, the cohort entry date was the date of the first registered SAD diagnosis. Unexposed individuals were assigned the same cohort entry date as their matched exposed counterparts.”* | |  |
|  |  |  |  |  |  | (*e*) Describe any sensitivity analyses | | - | N/A | |  |
| Results | | | | | | | | | | | |
| Participants | | | | | 13 | (a) Report numbers of individuals at each stage of study—eg numbers potentially eligible, examined for eligibility, confirmed eligible, included in the study, completing follow-up, and analysed | | 7-8 | Matched cohort section (pages 7-8). | |  |
|  |  |  |  |  |  | (b) Give reasons for non-participation at each stage | | 7-8 | Matched cohort section (pages 7-8). | |  |
|  |  |  |  |  |  | (c) Consider use of a flow diagram | | - | Supplementary figure 1. | |  |
| Descriptive data | | | | | 14 | (a) Give characteristics of study participants (eg demographic, clinical, social) and information on exposures and potential confounders | | 8 | Matched cohort section (pages 7-8) and Table 1. | |  |
|  |  |  |  |  |  | (b) Indicate number of participants with missing data for each variable of interest | | - | Table 1 | |  |
|  |  |  |  |  |  | (c) *Cohort study*—Summarise follow-up time (eg, average and total amount) | | - | Table 1 | |  |
| Outcome data | | | | | 15 | *Cohort study*—Report numbers of outcome events or summary measures over time | | 8 | Matched cohort section (pages 7-8) and Table 1. | |  |
|  |  |  |  |  |  | *Case-control study—*Report numbers in each exposure category, or summary measures of exposure | | - | N/A | |  |
|  |  |  |  |  |  | *Cross-sectional study—*Report numbers of outcome events or summary measures | | - | N/A | |  |
| Main results | | | | | 16 | (*a*) Give unadjusted estimates and, if applicable, confounder-adjusted estimates and their precision (eg, 95% confidence interval). Make clear which confounders were adjusted for and why they were included | | 7-10 | Results section, Tables 2-4 (Model 1). | |  |
|  |  |  |  |  |  | (*b*) Report category boundaries when continuous variables were categorized | | - | N/A | |  |
|  |  |  |  |  |  | (*c*) If relevant, consider translating estimates of relative risk into absolute risk for a meaningful time period | | - | N/A | |  |
| Other analyses | | | | | 17 | Report other analyses done—eg analyses of subgroups and interactions, and sensitivity analyses | | 9-10 | Results section (pages 9-10): sex-stratified analysis, adjusted analyses, sibling analyses. | |  |
| Discussion | | | | | | | | | |  |  |
| Key results | | | | | 18 | Summarise key results with reference to study objectives | | 10 | *“We investigated all-cause and cause-specific mortality in a cohort of over 50,000 individuals with SAD diagnosed in specialist services, compared with demographically-matched unexposed individuals. The risk of mortality in those with SAD was over two times higher than in the unexposed group, which was attributable primarily to unnatural causes of death, such as suicide, but also to natural causes of death, even after adjusting for a range of socioeconomic variables.”* | |  |
| Limitations | | | | | 19 | Discuss limitations of the study, taking into account sources of potential bias or imprecision. Discuss both direction and magnitude of any potential bias | | 13 | Limitations paragraph (page 13). | |  |
| Interpretation | | | | | 20 | Give a cautious overall interpretation of results considering objectives, limitations, multiplicity of analyses, results from similar studies, and other relevant evidence | | 13 | *“Individuals with SAD face an increased risk of mortality, particularly due to unnatural causes of death, such as suicide. Psychiatric comorbidities, particularly substance use disorders, significantly contribute to this excess death. Clarifying the underlying mechanisms may inform targeted prevention strategies and ultimately reduce mortality in this vulnerable population.”* | |  |
| Generalisability | | | | | 21 | Discuss the generalisability (external validity) of the study results | | 13 | Limitations paragraph (page 13). | |  |
| Other information | | | | | |  |  |  |  |  |  |
| Funding | | | | | 22 | Give the source of funding and the role of the funders for the present study and, if applicable, for the original study on which the present article is based | | 14 | *“Financial support: This study was supported by grants from Region Stockholm (ALF Medicin project grants; reference numbers 20160143 and 20180078), the Swedish Society of Medicine (Svenska Läkaresällskapets; reference number SLS-879801), and Karolinska Institutet (reference number FS-2018:0007), all awarded to Lorena Fernández de la Cruz.”* | |  |
