# Supplementary material for: All-cause and cause-specific mortality in social anxiety disorder: a matched cohort and sibling cohort study
Source: Epidemiol Psychiatr Sci. 2026 Mar 27;35:e13. doi: 10.1017/S2045796026100535 (PMC13122535; doi:10.1017/S2045796026100535)
Supplement: Fernández de la Cruz et al. supplementary material 2 — Fernández de la Cruz et al. supplementary material [file S2045796026100535sup002.docx]

**SUPPLEMENTARY MATERIAL**

**Supplementary Table 1.** Swedish International Classification of Diseases (ICD), 10^th^ edition codes used to group specific causes of death in the study.

| **Specific cause of death** | **Swedish ICD-10 diagnostic codes** |
| --- | --- |
| Certain infectious and parasitic diseases^a^ | A00−B99 |
| Neoplasms | C00−D48 |
| Diseases of the blood and blood-forming organs and certain disorders involving the immune mechanism^a^ | D50−D89 |
| Endocrine, nutritional, and metabolic diseases | E00−E90 |
| Mental and behavioral disorders | F00−F99 |
| Diseases of the nervous system | G00−G99 |
| Diseases of the eye and adnexa^a^ | H00−H59 |
| Diseases of the ear and mastoid process^a^ | H60−H95 |
| Diseases of the circulatory system | I00−I99 |
| Diseases of the respiratory system | J00−J99 |
| Diseases of the digestive system | K00−K93 |
| Diseases of the skin and subcutaneous tissue^a^ | L00−L99 |
| Diseases of the musculoskeletal system and connective tissue^a^ | M00−M99 |
| Diseases of the genitourinary system | N00−N99 |
| Pregnancy, childbirth, and the puerperium^a^ | O00−O99 |
| Certain conditions originating in the perinatal period^a^ | P00−P96 |
| Congenital malformations, deformations and chromosomal abnormalities^a^ | Q00−Q99 |
| Symptoms, signs and abnormal clinical and laboratory findings, not elsewhere classified | R00−R99 |
| Codes for special purposes^a^ | U00-U99 |
| External causes of morbidity and mortality | V01−Y98  **Accidents:**  V01-X59  **Suicide**:  X60-X84, Y10-34 |

^a^Grouped together under ‘Other natural causes of death’ given the small number of deaths due to this cause in the study (main matched cohort analysis).

**Supplementary Table 2.** Swedish International Classification of Diseases (ICD) codes used to identify lifestime diagnoses of psychiatric disorder comorbidities in the study.

| **Psychiatric disorders** | **Corresponding Swedish ICD-8 diagnostic codes** | **Corresponding Swedish ICD-9 diagnostic codes** | **Corresponding Swedish ICD-10 diagnostic codes** |
| --- | --- | --- | --- |
| **Neurodevelopmental disorders (attention-deficit/ hyperactivity disorder^a^, pervasive developmental disorders, and Tourette syndrome and chronic tic disorder^b^)** | 306.3 | 299, 307C, 314 | F84, F90, F95 |
| **Schizophrenia, other psychotic disorders** | 295 (minus 295.5), 297, 298 (minus 298.09) | 295 (minus 295F),  297, 298 (minus 298A) | F20, F21, F22, F23, F24, F25 (minus F25.0), F28, F29 |
| **Bipolar disorders** | 296 (minus 296.0 and 296.2) | 296A, 296C, 296D, 296E, 296W, 296X | F25.0, F30, F31, F34.0 |
| **Depressive disorders (major depressive disorder, persistent mood disorder, and unspecified mood disorder)** | 296.0, 296.2, 298.09 | 296B, 298A, 300E, 311 | F32, F33, F34 (minus F34.0), F38, F39 |
| **Other anxiety disorders (phobic, anxiety, obsessive-compulsive disorder, reaction to severe stress, and adjustment disorders)** | 300.00, 300.20, 300.3,307, 308.4 | 300A, 300C, 300D, 308, 309 | F40 (minus F40.1), F41, F42, F43 |
| **Eating disorders** | - | 307B, 307F | F50 |
| **Substance use disorders** | 303, 304 | 303, 304, 305A, 305X | F10-F16 and F18-19 |

^a^Individuals with attention-deficit/hyperactivity disorder (ADHD) were also identified by prescription of ADHD drugs, collected from the Prescription Drug Register, specifically Amphetamine (Anatomical Therapeutic Chemical [ATC] Classification System code: N06BA01), Dexamphetamine (N06BA02), Methylphenidate (N06BA04), Atomoxetine (N06BA09), and Lisdexamphetamine (N06BA12). ^b^Tourette syndrome and chronic tic disorder were identified following the algorithm described in Rück et al. (2015)^.^

**Supplementary Table 3.** Hazard ratios (HRs) with 95% confidence intervals (CIs) for all-cause mortality, natural, and unnatural causes of death among females with social anxiety disorder, compared to matched unexposed females.

|  | **Females with social anxiety disorder**  **(N=32,493)** | **Matched unexposed females**  **(N=324,930)** | **HR (95% CI)**  **Model 1, minimally adjusted^a^** | **HR (95% CI)**  **Model 2, additionally adjusted for socioeconomic variables^b^** |
| --- | --- | --- | --- | --- |
| **Causes of death** | **n (%)** | **n (%)** |  |  |
| **All-cause mortality** | 842 (2.59) | 3,195 (0.98) | **2.78 (2.58-3.00)** | **2.07 (1.90-2.24)** |
| **Natural causes of death** | 552 (1.70) | 2,795 (0.86) | **2.06 (1.88-2.26)** | **1.63 (1.48-1.79)** |
| Neoplasms | 202 (0.62) | 1,452 (0.45) | **1.40 (1.21-1.62)** | **1.25 (1.07-1.46)** |
| Endocrine, nutritional, and metabolic diseases | 24 (0.07) | 83 (0.03) | **2.87 (1.82-4.52)** | **1.90 (1.16-3.12)** |
| Mental and behavioral disorders | 12 (0.04) | 57 (0.02) | **2.08 (1.12-3.87)** | 1.74 (0.90-3.34) |
| Diseases of the nervous system | 27 (0.08) | 136 (0.04) | **2.00 (1.33-3.03)** | 1.54 (0.99-2.40) |
| Diseases of the circulatory system | 127 (0.39) | 501 (0.15) | **2.55 (2.10-3.10)** | **1.80 (1.47-2.22)** |
| Diseases of the respiratory system | 61 (0.19) | 179 (0.06) | **3.41 (2.55-4.57)** | **2.56 (1.87-3.52)** |
| Diseases of the digestive system | 43 (0.13) | 120 (0.04) | **3.53 (2.49-5.00)** | **2.34 (1.61-3.41)** |
| Symptoms, signs and abnormal clinical and laboratory findings, not elsewhere classified | 25 (0.08) | 95 (0.03) | **2.61 (1.68-4.06)** | **2.04 (1.26-3.31)** |
| Other natural causes of death^c^ | 31 (0.10) | 172 (0.05) | **1.79 (1.22-2.63)** | 1.50 (1.00-2.24) |
| **Unnatural causes of death** | 290 (0.89) | 400 (0.12) | **7.24 (6.22-8.42)** | **4.21 (3.56-4.99)** |
| Accidents | 73 (0.22) | 138 (0.04) | **5.26 (3.96-6.99)** | **3.07 (2.23-4.24)** |
| Suicides | 213 (0.66) | 247 (0.08) | **8.54 (7.10-10.3)** | **4.99 (4.06-6.12)** |

*Note:* Significant estimates are highlighted in bold. ^a^ Adjusted for the matching variables (i.e., birth year and county of residence at the time of the social anxiety disorder diagnosis). ^b^ Adjusted for the matching variables, country of birth (Sweden *vs.* abroad), and latest recorded highest level of education, family income level, and civil status. ^c^ Includes all groups with a small number of deaths (≤10) and the causes of death classified in the ICD as ‘codes for special purposes’.

**Supplementary Table 4.** Hazard ratios (HRs) with 95% confidence intervals (CIs) for all-cause mortality, natural, and unnatural causes of death among males with social anxiety disorder, compared to matched unexposed males.

|  | **Males with social anxiety disorder**  **(N=24,867)** | **Matched unexposed males**  **(N=248,670)** | **HR (95% CI)**  **Model 1, minimally adjusted^a^** | **HR (95% CI)**  **Model 2, additionally adjusted for socioeconomic variables^b^** |
| --- | --- | --- | --- | --- |
| **Causes of death** | **n (%)** | **n (%)** |  |  |
| **All-cause mortality** | 1,513 (6.08) | 4,605 (1.85) | **3.55 (3.34-3.76)** | **2.33 (2.19-2.49)** |
| **Natural causes of death** | 756 (3.04) | 3,600 (1.45) | **2.22 (2.05-2.40)** | **1.55 (1.43-1.69)** |
| Certain infectious and parasitic diseases | 12 (0.05) | 73 (0.03) | 1.62 (0.88-2.99) | 0.90 (0.47-1.71) |
| Neoplasms | 169 (0.68) | 1,356 (0.55) | **1.24 (1.06-1.46)** | 1.13 (0.96-1.33) |
| Endocrine, nutritional, and metabolic diseases | 29 (0.12) | 140 (0.06) | **2.05 (1.37-3.06)** | 1.20 (0.78-1.84) |
| Mental and behavioral disorders | 54 (0.22) | 97 (0.04) | **5.58 (4.00-7.79)** | **3.41 (2.34-4.98)** |
| Diseases of the nervous system | 31 (0.12) | 169 (0.07) | **1.83 (1.25-2.68)** | 1.19 (0.78-1.80) |
| Diseases of the circulatory system | 266 (1.07) | 1,091 (0.44) | **2.50 (2.18-2.86)** | **1.67 (1.44-1.93)** |
| Diseases of the respiratory system | 48 (0.19) | 194 (0.08) | **2.47 (1.80-3.39)** | **1.64 (1.17-2.31)** |
| Diseases of the digestive system | 85 (0.34) | 205 (0.08) | **4.11 (3.19-5.29)** | **2.29 (1.73-3.02)** |
| Symptoms, signs and abnormal clinical and laboratory findings, not elsewhere classified | 45 (0.18) | 146 (0.06) | **3.03 (2.17-4.23)** | **1.73 (1.18-2.53)** |
| Other natural causes of death^c^ | 17 (0.07) | 129 (0.05) | 1.31 (0.79-2.17) | 0.72 (0.42-1.24) |
| **Unnatural causes of death** | 757 (3.04) | 1,005 (0.40) | **7.52 (6.84-8.27)** | **4.21 (3.78-4.68)** |
| Accidents | 298 (1.20) | 443 (0.18) | **6.62 (5.71-7.66)** | **3.32 (2.81-3.94)** |
| Suicides | 442 (1.78) | 512 (0.21) | **8.63 (7.59-9.81)** | **5.14 (4.45-5.94)** |
| Other unnatural causes of death | 17 (0.07) | 50 (0.02) | **3.37 (1.95-5.85)** | **2.55 (1.39-4.69)** |

*Note:* Significant estimates are highlighted in bold. ^a^ Adjusted for the matching variables (i.e., birth year and county of residence at the time of the social anxiety disorder diagnosis). ^b^ Adjusted for the matching variables, country of birth (Sweden *vs.* abroad), and latest recorded highest level of education, family income level, and civil status. ^c^ Includes all groups with a small number of deaths (≤10) and the causes of death classified in the ICD as ‘codes for special purposes’.
